# Bacterial cytoplasm as an effective cell compartment for producing functional VHH-based affinity reagents and *Camelidae* IgG-like recombinant antibodies

**DOI:** 10.1186/s12934-014-0140-1

**Published:** 2014-09-16

**Authors:** Selma Djender, Aurelie Schneider, Anne Beugnet, Ronan Crepin, Klervi Even Desrumeaux, Chiara Romani, Sandrine Moutel, Franck Perez, Ario de Marco

**Affiliations:** Tumor Target and Therapeutic Antibody - Identification Platform (TAb-IP), Institut Curie, 3-5 Impasse Reille, 75014 Paris, France; Angelo Nocivelli Institute of Molecular Medicine, Division of Gynecologic Oncology, University of Brescia, Brescia, Italy; UMR144, Institut Curie, 12 Lohmond, 75005 Paris, France; Translational Research Department, Institut Curie, 26 rue d’Ulm, F75248 Paris Cedex 05, France; SIRIC INCa-DGOS-4654, Paris, France; CIC IGR Curie 1428, Paris, France; Department of Biomedical Sciences and Engineering, University of Nova Gorica (UNG), Glavni Trg 9 - SI-5261, Vipava, Slovenia

**Keywords:** Affinity purification, Avidity effect, Disulfide bonds, Immune diagnostics, Single-chain antibodies, Single-domain antibodies, Sortase, Sulfhydryl oxidase

## Abstract

**Background:**

The isolation of recombinant antibody fragments from displayed libraries represents a powerful alternative to the generation of IgGs using hybridoma technology. The selected antibody fragments can then be easily engineered into (multi)-tagged constructs of variable mass and complexity as well as reconstituted into *Camelidae* IgG-like molecules when expressed fused to Fc domains. Nevertheless, all antibody constructs depend on an oxidizing environment for correct folding and consequently still belong to the proteins difficult to express in bacteria. In such organisms they are mostly produced at low yields in the periplasmic space.

**Results:**

We demonstrate that fusion constructs of recombinant antibodies in combination with multiple tags can be produced at high yields and totally functional in the cytoplasm of bacteria expressing sulfhydryl oxidase. The method was applied to structurally demanding molecules such as VHHs fused to SNAP and Fc domains and was validated using the antibody-derived reagents in a variety of immune techniques (FACS, ELISA, WB, IP, SPR, and IF).

**Conclusions:**

The collected data demonstrate the feasibility of a method that establishes a totally new approach for producing rapidly and inexpensively functional *Camelidae* IgG-like monoclonal antibodies and antibody-based reagents containing multiple disulfide bonds and suitable for both basic research and clinical applications.

**Electronic supplementary material:**

The online version of this article (doi:10.1186/s12934-014-0140-1) contains supplementary material, which is available to authorized users.

## Background

Monoclonal antibodies are probably the most versatile and widespread class of reagents used in biology research, diagnostics, and therapy. Although IgG antibodies recovered from hybridoma cells still dominate the field, alternative formats know a constantly increasing success and recombinant Fab, single-chain (scFv) and single-domain (VHH) antibodies have demonstrated their reliability in basic research as well as in clinical applications [1,2]. In the case of VHHs, their reduced mass is a decisive advantage to obtain faster clearance for *in vivo* imaging, a better penetration in solid tumors and even the permeation across the blood brain barrier [3-5]. The *in vitro* selection allows for the isolation of binders for toxic or scarcely antigenic targets as well as for epitopes correlated to specific functions. Whole cells have been successfully used for panning antibody fragments that recognize membrane proteins in their native membrane environment and for identifying new biomarkers [6]. Basic molecular biology techniques allows for VHH fusion to tags and larger carriers to obtain application-optimized reagents [7]. Single-domains can be easily reconstituted into the *Camelidae* IgG-like format by fusion to a Fc domain and Fc moieties with different characteristics can be selected to tune ADCC and CDC effects in different organisms.

In contrast to conventional antibodies, recombinant antibodies are routinely expressed also in prokaryotic systems. Bacteria can be used to display on their surface antibodies of different format for diagnostic applications [8,9] and to obtain elevated VHH productions in both the periplasm and the cytoplasm [10-13]. In contrast, the yields of reconstituted IgG-like molecules and fusions with some valuable tags remain low [14,15] due to either structural complexity or different redox requirements of the two partner polypeptides. Recently, it has been demonstrated that the cytoplasmic co-expression of disulfide-bond dependent proteins together with sulfhydryl oxidase and a disulfide bond isomerase increased significantly the production of the target proteins [16]. Such approach proved being effective to improve the cytoplasmic accumulation of full-length VHH-SNAP tag fusions [17]. Nevertheless, no proof of antibody functionality was shown in this preliminary communication. Now we demonstrate that several VHH-based constructs as complex as the IgG-like reconstituted VHH-Fc antibodies can be produced in bacterial cytoplasm at elevated yields and preserve their complete functionality. This opportunity represents a time and cost effective alternative to the conventional expression of IgG antibodies from hybridoma cells. Furthermore, it allows the production of fusion molecules such as the VHH-SNAP or VHH-GFP constructs that are difficult to obtain in oxidizing environments.

## Results and discussion

The *in vitro* recovery of recombinant antibodies represents an effective and rapid alternative for isolating binders against any antigen class. Furthermore, the access to the antibody sequence simplifies molecular engineering and opens the possibility to fuse suitable tags to the antibodies to develop them in reagents optimally suited to different applications. Such fusion constructs are often easy to produce in bacteria and we designed a collection of vectors for the parallel expression of application-friendly VHHs production in both bacterial periplasm and cytoplasm (Additional file 1: Figure S1). In a preliminary expression test we noticed that tags could significantly modify the antibody stability and yields. SNAP/CLIP and GFP were poorly folded and were prone to aggregate when expressed in the periplasm whereas the presence of t*rans* disulfide bonds in structurally complex proteins such as alkaline phosphatase, peroxidases, and enzymatic toxins seemed to be compatible only with periplasmic expression. We designed a decision chart with the aim of optimizing the cytoplasmic expression of fusion antibodies that either failed to be expressed in the periplasm or, such as the Fc-fusions, accumulated in low amounts (Additional file 2: Figure S2). Bacterial mutants in which the cytoplasmic reducing metabolism is impaired have been sometimes successfully used to express disulfide-dependent proteins but the results are contradictory specially when molecules with multiple disulfide bonds must be produced [11,18]. Therefore, we evaluated the promising alternative of inducing the formation of disulfide bonds in the cytoplasm of wild type bacteria in which a recombinant eukaryotic sulfhydryl oxidase is accumulated [16]. The expression of the oxidizing enzyme is triggered by arabinose addition to the culture medium and can be induced independently and before the expression of the antibodies by means of IPTG addition.

**Figure 1:**
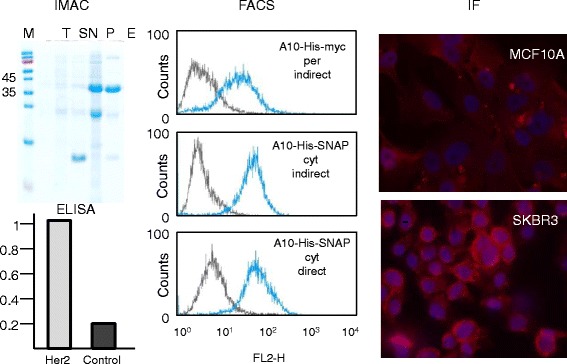
**Functional validation of the anti-HER2 SNAP-tagged VHH A10 expressed in the bacterial cytoplasm.** Cytoplasmic-expressed A10-SNAP fusion protein was eluted at almost homogeneity (E) after a single metal affinity chromatographic (IMAC) step. Both a portion of the fusion protein and SNAP alone accumulated in the pellet (P). FACS analysis (filter band-pass: 564-606 nm) was performed using HER2 negative (MCF10A) and positive (SKBR3) cells and used to compare different A10 constructs and detection methods: i) myc + His-tagged antibodies expressed in bacterial periplasm in combination with anti-His and a secondary antibodies; ii) SNAP + His-tagged antibody expressed in bacterial cytoplasm in combination with anti-His plus secondary antibody; iii) SNAP + His-tagged antibody expressed in bacterial cytoplasm directly linked to the SNAP-Surface 549 (NEB) chromophore. The same cell lines were used in combination with A10-SNAP for cell ELISA and for immunostaining HER2 at the cell membrane of negative (MCF10A) and positive (SKBR3) cells.

**Figure 2:**
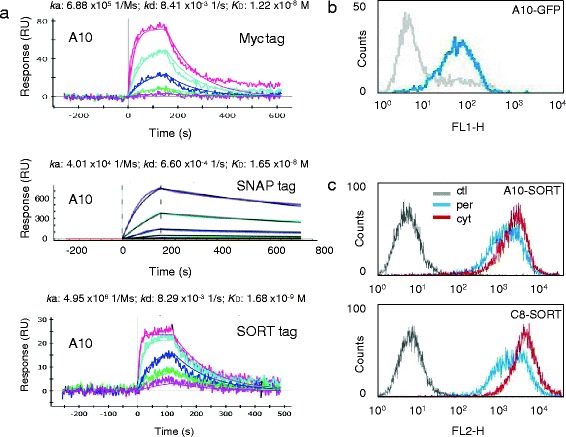
**Comparison between antibody constructs expressed in the bacterial cytoplasm and periplasm. a)** The affinity of the VHH A10 for its antigen HER2 (purified ectodomain) was measured by Surface Plasmon Resonance. Three constructs were compared: A10 tagged myc + His and expressed in the bacterial periplasm, A10 tagged SNAP + His and A10 tagged SORT + His, both expressed in the bacterial cytoplasm. The graphs correspond to a single experiment representative of at least three repeats. The analyte concentrations were in the range 300-3.5 nM (myc-tag), 100-3.5 nM (SNAP-tag), and 30-0.35 nM (SORT-tag). **b)** The anti-HER2 antibodies A10 fused to GFP was expressed in the cytoplasm and directly assessed by FACS (filter band-pass: 515-545 nm) using HER2 negative (MCF10A) and positive (SKBR3) cells. **c)** The anti-HER2 antibodies A10 and C8 were expressed fused to the LPTEG peptide (SORT tag) in both the periplasm and the cytoplasm and the resulting samples were assessed by FACS (filter band-pass: 564-606 nm) using HER2 negative (MCF10A) and positive (SKBR3) cells, and the 6xHis tag for visualization.

By panning a pre-immune library, we isolated several VHHs specific for the ectodomain of the human HER2 receptor (data not shown) and expressed three of them (A10, C8, G3) using both the original periplasmic phagmid vector (pHEN2) which allows for the fusion to a His-myc tag and a cytoplasmic vector (pSNAP) for obtaining a His-SNAP fusion. The constructs were purified by IMAC and successively by gel filtration for separating aggregates and polymerization products (Additional file 3: Figure S3a). The yields of the SNAP-fusions were significantly higher than those of the myc-fusions (Table 1) and the degradation products/aggregates represented a negligible percentage of the affinity-purified antibodies (Figure 1 and Additional file 3: Figure S3a). We then compared the performance of the constructs expressed in the periplasm (His-myc, per) and cytoplasm (His-SNAP, cyt) at the FACS. In a first experiment, the detection was obtained in both cases using an anti-His labeled secondary antibody (indirect labeling). The A10-SNAP construct discriminated the HER2-positive SKBR3 and MCF10A negative cell populations even better than the A10-myc (Figure 1). However, the advantage of the SNAP tag is that it can be covalently labeled before immune detection (FACS or IF) enabling the direct detection of the antigen. The construct A10-SNAP coupled to the chromophore SNAP-Surface 549 successfully identified its antigen with no signal loss in FACS (Figure 1). The A10 clone fused to SNAP showed specificity in cell ELISA and in immunofluorescence when HER2-positive SKBR3 and HER2-negative MCF10A cells were compared (Figure 1). A10 myc and SNAP constructs showed comparable SPR-measured affinities for the purified ectodomain of HER2, a further clear indication that the cytoplasmic expression did not decrease the functionality of the recombinant antibody fused to the SNAP tag (Figure 2a). This first set of results demonstrated that the cytoplasmic expression of VHH-SNAP fusions in the presence of sulfhydryl oxidase enabled to produce functional antibodies the binding capacity of which was at least as good as that of the same VHHs expressed in the periplasm, but with significantly higher yields. Similarly, we successfully expressed, labeled, and validated by FACS a scFv-SNAP construct (anti-claudin-3, Additional file 4: Figure S4a), the A10 VHH fused to the tags CLIP and HALO (Additional file 4: Figure S4b), and produced a VHH-GFP fusion protein that provided a clear signal shift in flow cytometry without the necessity of using primary/secondary commercial antibodies (Figure 2b). Wild type GFP cannot be exported to the periplasm by the SEC pathway in an active form [19] and, although some fluorescent protein variants are able to accumulate in this oxidizing cell compartment necessary for the formation of the antibody disulfide bonds, the choice remains limited to few molecules [20,21]. Here we demonstrate the feasibility of an approach to produce in the bacterial cytoplasm fluorescent immune reagents that should be not restricted to few specific sequences.

**Table 1:** **Yield comparison of anti-HER2 VHHs fused together to different tags**

	**A10 mg/L**	**C8 mg/L**	**G3 mg/L**
Myc per IMAC	1-5	1-5	<1
Myc per SEC	1-2	<1	<0.5
SNAP cyt IMAC	15-20	20-25	10-15
SNAP cyt SEC	15-20	20-25	10-15
SORT-Tag cyt IMAC	6-8	4-7	4-7
SORT-Tag cyt SEC	4-6	3-5	3-5
SORT-Tag per IMAC	<0.1	<0.1	<0.1
Fc cyt IMAC	25-30	10-15	15-20
Fc cyt SEC	25-30	10-15	10-15

The recombinant antibodies were cloned in vectors that promote either periplasmic (per) or cytoplasmic (cyt) accumulation and folding, expressed in bacteria, and finally purified by metal affinity chromatography (IMAC) followed by gel filtration (Size Exclusion Chomatography - SEC). The yields are expressed in mg/L of culture and indicate the range obtained in at least three independent purification procedures.

In these first examples of cytoplasmic antibody expression, no direct comparison between the same construct expressed either as cytoplasmic or secreted protein was possible because we did not manage to recover soluble VHH-GFP and VHH-SNAP fusion proteins from the bacterial periplasm. Therefore, we used another tag that could be expressed in both bacterial cell compartments to obtain comparable samples. The SORT-tag is a short peptide (LPTEG) that allows for the sortase-dependent covalent conjugation to N-terminal available glycine residue(s) [22] and has been used for obtaining fusions between several classes of molecules [23,24]. Surprisingly, the presence of this short tag instead of the conventional myc strongly decreased the solubility of all three tested anti-HER2 antibodies when they were expressed in the periplasm (Table 1). In contrast, the cytoplasmic yields were elevated and, in terms of molarity, comparable with those obtained using SNAP as a fusion tag (Table 1). The SORT-tagged antibodies produced in the periplasm and in the cytoplasm labeled at similar intensity HER2 pos and HER2 neg cells in FACS experiments, with a reproducible stronger signal for the cytoplasmic constructs (Figure 2c). Unexpectedly, the cytoplasmic A10-SORT construct showed higher SPR-measured affinity for its antigen than the control A10-myc construct recovered from the periplasm (Figure 2a). The difference was totally due to the improved association rate. This result confirmed the binding functionality of the construct expressed in the cytoplasm and suggests that despite having similar overall mass, the relative length and/or folding of the different tags can influence the accessibility and actual binding of the two constructs to the antigen epitope, as already noticed by other authors [25,26]. We cannot rule out that the small observed differences in terms of antibody binding capacity were due to minimal stability variation of the different constructs. If this is the case, cytoplasmic expression resulted more convenient than the periplasmic production of the same construct because it enabled both higher yields and slightly but repeatedly measured “improved” functionality.

Recombinant antibodies with tags such as SNAP/CLIP/HALO/GFP and SORT are suitable for several attractive biological applications, but still represent a niche in comparison to IgG antibodies. In some cases, introducing new protocols based on non-conventional antibody formats for performing immunological analyses might be demanding for the lab management. Therefore, it can be more practical to reconstitute antibody fragments into IgG-like molecules that can be used and detected as conventional monoclonal antibodies. Consequently, we expressed in the cytoplasm the same anti-HER2 set of antibodies used in the previous experiments but as fusions with a rabbit Fc domain. This tag offers some specific advantages such as the possibility to affinity purify the molecules using Protein A/G, to increase the avidity for the antigen by inducing dimerization, and to perform multiple parallel staining by exploiting Fc domains issued from different organisms [27].

**Figure 3:**
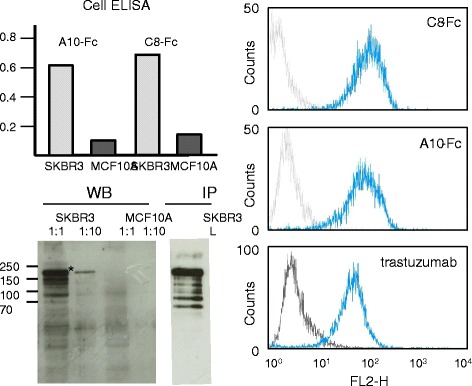
**Functional validation of Fc-VHH fusion antibodies.** Fc-VHH constructs corresponding to the clones A10 and C8 were expressed in the bacterial cytoplasm, purified, and finally tested by cell ELISA and compared with the monoclonal anti-HER2 therapeutic monoclonal antibody trastuzumab by FACS (filter band-pass: 564-606 nm) using HER2 negative (MCF10A) and positive (SKBR3) cells. C8-Fc was used for HER2 detection by WB and to immunoprecipitate HER2 from SKBR3 cells.

**Figure 4:**
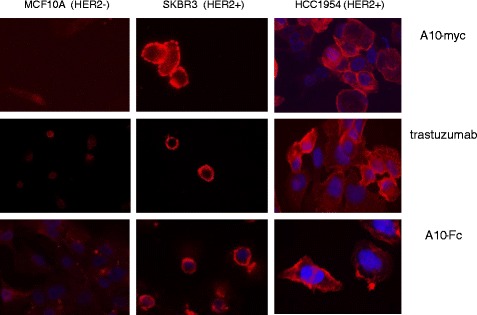
**Identification of the membrane receptor HER2 by immunofluorescence.** The IF staining pattern of the VHH A10 fused to either myc (periplasmic expression) or Fc (cytoplasmic expression) was compared with that of trastuzumab in HER2 neg and HER2 pos cells.

All the constructs were well expressed (Table 1) and highly pure already after IMAC step (Additional file 3: Figure S3b). Size exclusion chromatography (SEC) confirmed that the antibodies were monodispersed (Additional file 3: Figure S3b). The functionality of the bivalent Fc-VHHs was confirmed by cell ELISA and FACS. In this last case, the reconstituted IgG-like molecules were at least as effective as the therapeutic antibody trastuzumab (Figure 3). Although VHHs usually bind to conformational epitopes that are lost after antigen denaturation, C8 recognized its antigen by western blot in a total cell lysate and immunoprecipitated the full-length as well as many of the shedding products [28] of the HER2 ectodomain (Figure 3). These results indicate that the epitope recognized by the binder is probably very basal and that C8 could be useful for evaluating the metal protease-dependent HER2 isoform distribution in different cell populations. The IF staining pattern on HER2 positive and negative cells was identical for the monoclonal trastuzumab, the cytoplasmic produced IgG-like A10-Fc construct, and the monovalent periplasmic produced A10-myc construct (Figure 4).

**Figure 5:**
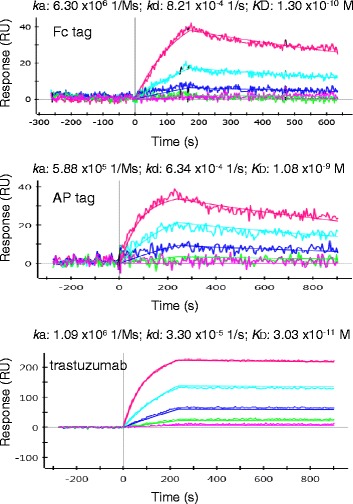
**Avidity effect of the bivalent Fc-VHH construct.** The avidity of the bivalent A10-Fc was compared with that of the bivalent constructs A10-alkaline phosphatase (AP, periplasmic bacterial expression) and trastuzumab (hybridoma expression). The graphs correspond to a single experiment representative of at least three repeats. The analyte concentrations were in the range 1-0.01 nM (myc-tag), 300-3.5 nM (AP-tag), and 1-0.01 nM (SORT-tag).

The measurement of the apparent affinity (avidity) of bivalent constructs confirmed that the tag characteristics can influence the antigen/antibody binding, as previously observed for the monovalent constructs (Additional file 5: Table S1). The avidity effect enabled the construct A10-Fc to bind its antigen one log stronger (Figure 5) than the monovalent constructs (Figure 2c). At the same time, there is a substantial difference between the avidities of the Fc and alkaline phosphatase (AP) fusions (Figure 5). Although expressed in the periplasm, the dimer A10-AP bound HER2 with a *K*_D_ in the lower nanomolar range, a log worse than the A10-Fc construct produced in the cytoplasm (Figure 5). This result confirms that functional binding capacity is not a simple matter of active domain number [29].

**Figure 6:**
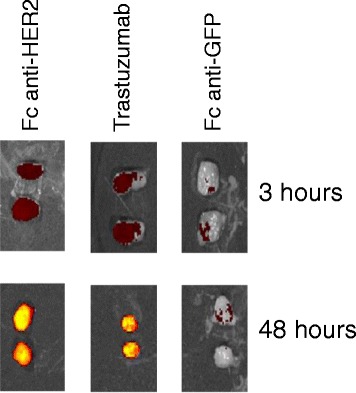
**Antibody accumulation in xenografted tumors.** HER2 positive tumors were recovered from treated mice 3 and 48 hours after antibody injection. Tumors were cut in two pieces along their median axis and their infrared fluorescence intensity detected at 770 nm.

Finally we evaluated the capacity of the IgG-like reconstituted antibodies produced in the bacterial cytoplasm to target a HER2-positive tumor *in vivo* using a xenografted murine model. C8 was preferred because it was the only of the three characterized antibodies that worked in both WB and IP and, therefore, we expected that it could recognize its antigen in a wider range of structural conformations. Animals were treated with infra-red labeled anti-HER2 C8-Fc and trastuzumab (positive control), as well as with an anti-GFP antibody (negative control). Tumors were recovered after both 3 and 48 hours, sectioned along the median axis to have access to the inner section, and the emitted infrared fluorescence was recorded (Figure 6). The mass (80 kDa) of reconstituted IgG-like Abs impairs direct kidney filtration and the rapid clearence specific of monovalent VHHs (14 kDa) [5,30]. Consequently, C8-Fc accumulated in the tumor tissues with the same kinetic of the positive control trastuzumab. Although fluorescence does not allow for accurate quantitative comparisons, the recombinant antibodies seem as effective as trastuzumab in concentrating inside the tumor. The tumors treated with the control anti-GFP recombinant antibody did not show any fluorescence signal at both 3 and 48 hours.

## Conclusions

Single-domain antibodies can be produced at elevated yields and their structural stability allows for the accumulation of functional molecules even in the cytoplasm of wild-type bacteria [13]. Here we show that even formats as complex as *Camelidae* IgG-like structures can be produced rapidly and cost-effectively in bacteria. Functional IgG recombinant antibodies have been already successfully produced in bacterial periplasm in the past [14] but yields of correctly assembled full-length molecules rarely exceeded 1 mg/L when shake flasks were used. This condition limited antibody discovery programs that rely on the availability of large batches of antibodies. Recently, engineered systems based on mutagenized over-expressing strains and optimized plasmids succeeded in yielding up to 4 mg/L of IgGs [15] and similar yields were obtained expressing scFv-Fc fusions in *Leishmania tarantolae* [31]. The protocol described in this article shows that it is possible to increase the yields to tens of mg/L and, according to recently published data [13], there is still the possibility to enhance the yields per liter of culture by increasing the cell density using optimized media and fermentation conditions. However, the main achievement of our approach is the demonstration –obtained by means of several independent techniques [32]- that the functionality of the IgG-like constructs is totally preserved when they are purified after expression in the cytoplasm of bacteria co-expressing sulfhydryl oxidase and the bacterial protein disulfide isomerase DsbC. The combined oxidative and isomerase activities apparently prevent the accumulation of non-functional antibodies. When the comparison was possible, the constructs expressed in the cytoplasm showed both lower aggregation and degradation than those measured in the periplasm. This approach, initially proposed by Ruddock’s group for producing disulfide-bond dependent secreted proteins [16,33], perfectly fits to antibody fragments fused to tags with different folding requirements [17,34]. The bottleneck represented by the saturation of the SEC-translocon [35] would explain the remarkable higher yields obtained when the antibodies were produced in the cytoplasm rather than in the periplasm. Lately we expressed tens of other VHH-Fc constructs using both rabbit and human Fc and clearly remarked yield difference among antibodies. This condition is probably due to the intrinsic stability of the different sequences but the cytoplasmic expression was constantly more productive than the periplasmic.

The present trend in both academic and industrial labs seems to be focused on using single domain antibodies from *Camelidae* and human origin as well as Fab fragments to identify new reagents and therapeutic molecules [36,37]. We believe that our approach might represent a break-through technology for researchers who need to produce significant amounts of antibodies and antibody-based reagents for preliminary *in vitro* and *in vivo* characterization. We produced the described antibodies in wild type *E. coli* that cannot provide eukaryotic-like glycosylation but our results should encourage expressing IgG-like constructs in modified bacteria [38] with the aim of obtaining antibodies with native post-translational modifications.

## Methods

### Plasmid preparation

pET14b-rFc. The rabbit Fc fragment of the pFUSE-rFc2-adapt-scFv vector [39] was amplified by PCR using forward primer GAGGCGGCCGCTAGATCTAGCAAGCCCA CGTG and backward primer GAGATTGGATCCATCATGTCTGGCCAGCTAGC. The PCR product was purified by agarose gel electrophoresis and sub-cloned into pET14b-hFc-multitag between *Not*I and *BamH*I. VHHs were then inserted in pET14b-rFc between *Nco*I and *Not*I.

pSORTp. The DNA sequence corresponding to GSSGG-LPETG-6xHis was synthesized by Proteogenix (France) and sub-cloned into the pHEN8 [40] vector using *Not*I and *Bsm*I. VHHs were then inserted in pSORTp between *Nco*I and *Not*I. The sequence of the VHH-GSSGG-LPETG-6xHis in the pSORTp vector was digested with *Nco*I and *BamH*I and sub-cloned into pET14b to obtain the cytoplasmic version of the vector (pSORTc).

The vector for the production of VHH and scFv antibodies fused to the SNAP tag used in this work is identical to the one described previously [7], but the lecture frame has been modified by introducing a further base to render it universal. CLIP and HALO sequences were substituted to SNAP sequence to yield the corresponding expression vectors.

### Antibody production

Recombinant antibodies were expressed in *E. coli* BL21 (DE3) strain transformed with a plasmid for the expression of sulfhydryl oxidase and DsbC [16,17]. Briefly, 2 mL of over-night preculture were used to inoculate 500 mL of LB broth in the presence of the appropriate antibiotics. For small-scale screening purpose, 30 μL were used to inoculate 3 mL of culture medium. Bacteria were grown at 37°C until OD600_nm_ reached 0.4. Sulfhydryl oxidase and DsbC expression was induced by adding 0.5% (g/mL culture) of arabinose and the temperature was lowered to 30°C. After 45 min, 0.05 mM of IPTG were added to induce antibody expression, the bacteria were grown overnight at 20°C, harvested, and frozen. Pellets were resuspended in 20 mL of 100 mM Tris pH8, 500 mM NaCl, 2.5 mM MgCl_2_. Lysozyme (0,5 mg/mL) and DNase (3U) were added and the lysate was kept 30 min at room temperature. Samples were sonicated and finally centrifuged at 18,000x*g* for 20 min at 4°C. Lysates were filtered and applied on either a HiTrap TALON® 5 mL column or on a HiTrap MabSelect SuRe 5 mL column in combination with a chromatographic AKTA pure system (GE Healthcare). Analytical [41] and preparative size exclusion chromatographic steps were performed on Superdex™ 75 5/150 and 10/300 GL, respectively (GE Healthcare). Antibody concentration was measured using BCA colorimetric assay. Denaturing SDS-PAGE gels (15%) were Coomassie stained and recorded using a Gel Doc™ EZ System (Bio Rad). In the case of small-scale production, the supernatant obtained after pellet lysis was incubated in the presence of Protein A sepharose beads (GE Healthcare), the beads were washed, resuspended in loading buffer, and the proteins separated by denaturing SDS-PAGE.

### Western blot and immunoprecipitation

Cell lysates were separated on a 12% SDS-PAGE and transferred to a nitrocellulose membrane. After 60 min in 5% milk-PBS, the membrane was incubated 1 h with 10 μg/mL of the C8-Fc (IgG-like) antibody specific for the HER2 ectodomain. After 3 washing steps in PBS, 4 μg/mL of anti-myc monoclonal Ab (9E10) were added followed by HRP-conjugated goat anti-mouse antibody (Cat. N. 32430, Pierce). HRP-dependent signal was identified by autoradiography using enhanced chemiluminescence.

For immunoprecipitation (IP), SKBR-3 cells were grown to 90% confluence in 150 mm culture dishes, washed twice with PBS, and their lysis was induced in the presence of 3 mL of 10 mM Tris–HCl, pH 8.0, 150 mM NaCl, 1% NP 40, 1 mM EDTA, protease inhibitor cocktail for each dish. After 1 h incubation at 4°C, the material was harvested by scraping and cell debris was separated from soluble lysate by centrifugation (10 min at 3600x*g* and 4°C). All the incubation steps were performed rocking the tubes constantly. Cleared cell lysates (5 mL) resuspend in an equal volume of IP buffer (10 mM Tris–HCl, pH 8.0, 150 mM NaCl, 1% NP40) were pre-incubated 1 h at 4°C in the presence of 200 μL of protein G agarose beads (Thermo Fisher) and successively washed 3 times in IP buffer to eliminate unspecific binding. The supernatant was recovered by centrifugation (3 min × 2500 *g*), mixed with 200 μg of the antibody, and incubated 2 h at 4°C. Finally, 200 μL of washed protein G agarose beads were added and washed after 1 h at 4°C 5 times in 10 mL of IP buffer five times before being resuspended in 50 μL of SDS loading buffer and heated 10 min at 95°C. The protein samples were fractionated on a 12% SDS-PAGE, transferred to a nitrocellulose membrane, and HER2 was identified by chemiluminescence as for the western blot.

### Flow cytometry analysis

Specific antibody binding to the HER2 receptor expressed at the cell surface was analyzed by flow cytometry using SKBR3 (positive sample) and MCF10A (negative sample) cells. Cells were first incubated in 96 well plates in the presence of 10% FCS-PBS to prevent unspecific binding and then 2 hours at 4°C cells with monovalent VHHs. Cells were analyzed with FACS-Calibur Flow Cytometer (BD Biosciences) after having incubated them 1 hour at 4°C with either a monoclonal anti-myc (9E10) or an anti-His (Tebu Bio) primary antibody followed by a goat anti-mouse antibody conjugated to phycoerythrin (BD Pharmingen). SNAP-tag was directly labeled using the chromophore SNAP-Surface AlexaFluor 549 (NEB).

The SNAP-Surface AlexaFluor 647 Protein Labeling kit (NEB) was employed to label the scFvH6-SNAP fusion protein according to the manufacturer’s instructions. Cell surface claudin-3 specific binding was tested on native unfixed OSPC-2 primary ovarian carcinoma cell line (positive sample) and human leukemia K562 cells (negative control). After detachment with 0.5 mM EDTA, cell viability was evaluated with Trypan Blue exclusion and only samples with more than 80% of viable cells were considered for staining. Membrane integrity of stained samples was assessed with PE Annexin V Apoptosis detection kit (BD Pharmingen) and AnnexinV negative viable cells where identified with an appropriate gating strategy. Approximately 2x10^5^ cells per test were used for each experiment. Cells were washed three times with PSB buffer supplemented with 0.5% BSA and incubated 1 hour at 20°C with 500 ng of scFvH6-Alexa 647. Cells were finally resuspended in PBS and analyzed by flow cytometry using FACS-Calibur and Cell quest Pro Software (BD). No commercial antibody for the claudin-3 ectodomain is available for direct comparison.

### ELISA

Assays were performed in 96 well round bottom plates. Wells were coated over-night at 4°C with either 5 μg/mL of recombinant HER2-Fc or 8 μg/mL of rabbit-IgG (Sigma) as a control. Wells were then saturated 2 hours at room temperature with 3% BSA-PBS and recombinant His-tagged VHHs were added at concentrations of 130 (Fc-constructs) and 303 (SNAP-constructs) nM. After 1 hour incubation at room temperature, the specific signal was visualized by incubating the samples 1 hour at room temperature in the presence of anti-His primary antibody (Tebu Bio) followed by a goat anti-mouse conjugated to peroxidase (Pierce). The OD was measured at 450 nm. Cell ELISA with VHH-Fc was performed using the same protocol and 10^5^ cells in suspension/well instead of purified HER2.

### Surface plasmon resonance analysis

The affinity and avidity of the anti-HER2 constructs for their substrate was measured at 25°C using a ProteOn XPR36 (BioRad). Fc-HER2 ectodomain produced in mammalian cells (96 kDa) was diluted to 2 μg/mL in sodium acetate buffer (pH 5.0) and immobilized by amine-coupling on a GLC chip (BioRad) at 2000 RU. 100 μL of antibodies were used as analyte and injected at 100 μL/min. The complete kinetic data set for each antibody was collected in a single run and fitted using a 1:1 Langmuir interaction model. Surface regeneration was performed using 10 mM glycine HCl, pH 2.0 (IgG-like format) or 3.0 (VHH format).

### Immunofluorescence

MCF10A and SKBR3 cells were seeded on coverslips overnight. The day after, cells were first incubated 15 min at room temperature in 4% PFA-PBS and then 2 hours at room temperature in the presence of the antibodies. Samples were incubated 45 min in the dark in the presence of suitable Cy3-conjugated antibodies and finally mounted on microscope slides by using Mowiol.

### Tumor targeting *in vivo*

Trastuzumab, the C8 single-domain fused to rabbit Fc (C8-Fc), and the anti-GFP control VHH were diluted to 1 mg/mL in PBS and incubated in the presence of CF770 NHS esters (IVIS Xenolight, Caliper) at a 7.2 dye-to-protein molar ratio according to the manufacturer’s protocol. Unreacted CF770 dye was removed by gel filtration using a NanoSep vial (IVIS Xenolight, Caliper). Labeled protein samples were stored in the dark at 4°C.

HER2 positive BC111 tumor model was grafted subcutaneously into the interscapular fat pad of female Swiss nude mice under anesthesia. Experimental protocol and animal housing were performed according to institutional and French Ethics Committee guidelines (Agreement B75-05-18, France). Xenografts of 0.6-1 mm developed two months after grafting, mice were anaesthetized with isoflurane and 25 (3 hour experiments) or 100 μg (48 hour experiments) of CF770 labeled antibodies in a total volume of 100 μL were injected intravenously in the tail vein. Infra-red fluorescence images were acquired using IVIS Spectrum equipment and analyzed with Living Image 3.2 software (Caliper Life Sciences).

## Additional files

Additional file 1: Figure S1. Application-friendly vectors for single-domain antibody expression. The vector set provides the possibility to fuse any antibody originated from a pHEN2 library to a double tag (6 x His plus specific tag) suitable for *ad hoc* applications. All the vectors have a conserved *Nco*I*-Not*I cloning site. Some tags are compatible for expression in both periplasm and cytoplasm whereas no conclusive data are available for other tags for which either cytoplasmic or periplasmic expression have been confirmed so far. SNAP (human O6-alkylguanine-DNA alkyltransferase); CLIP (modifid human O6-alkylguanine-DNA alkyltransferase); HALO (modified haloalkane dehalogenase); GFP (green fluorescent protein); SORT (amino acid sequence LPTEG for sortase-mediated covalent binding); BS (biotinylation sequence); cys (free cysteine for maleimide reactions); C-tag (amino acid sequence EPEA); Fc (Fc-domains, in our case rabbit and human subtype IgG2); toxin (any secreted toxin); AP (alkaline phosphatase); POX (peroxidase). Additional file 2: Figure S2. Production flow-chart for the different recombinant antibody constructs. According to the vector used for cloning, the plasmids conceived for producing tagged antibodies are transformed in *E. coli* strains with suitable features and the corresponding antibodies will accumulate either in the bacterial cytoplasm or periplasm. Lack of transformation can be the consequence of poor cell competence or misuse of the selection antibiotics (ABs). Colonies of transformed cells are used for both pre-culture inoculation and the preparation of glycerol stocks. Cytoplasmic expression is performed in bacteria co-expressing sulfhydryl oxidase and DsbC isomerase under the control of arabinose and the production of these folding-support enzymes is triggered before the IPTG-dependent expression of the target antibody. Periplasmic expression is performed in shorter time to prevent excessive product leakage into the culture medium. The addition of glucose is intended to suppress basal expression and high IPTG concentrations should enable rapid saturation of the expression machinery. Additional file 3: Figure S3. Quality control of the SNAP- and Fc-tagged anti-HER2 VHHs produced in the cytoplasm. SNAP-tagged antibodies were first affinity-purified by IMAC and their polydispersity was successively evaluated by analytical Size Extrusion Chromatography (SEC). The black bar corresponds to the exclusion volume. The proteins recovered in the fractions were finally separated by SDS-PAGE and visualized using colloidal blue. The A10 construct has been reported as an example.a) Fc-tagged antibodies were first expressed and purified in small-scale to assess the protein accumulation in the lysates (L) and bound to the beads after washing (B). Numbering indicates either contemporary expression induction of Fc-VHH and sulfhydryl oxidase (1) or to anticipated sulfhydryl oxidase expression (2). For large-scale production, the constructs were affinity-purified using Protein A and their polydispersity was successively evaluated by analytical SEC. The black bar corresponds to the exclusion volume. The protein was visualized after SDS-PAGE separation. The A10 and C8 constructs have been reported as examples. Additional file 4: Figure S4. Expression of fusion immunoreagents composed of antibody fragments and SNAP, CLIP, and HALO tags. a) The H6 scFv antibody specific for claudin-3 was fused to the SNAP-tag and the resulting construct labeled with Alexa 647 before FACS analysis (filter band-pass: 653-669 nm) using unfixed human ovary cancer line OSPC-2 that expresses claudin-3 and K562 cells as a negative control. Green lines indicate the level of reactivity of the scFvH6-SNAP, gray lines represent cells in the absence of the antibody. Percentage of claudin-3 postive cells is reported in each plot. b) Fusions of CLIP and HALO tags with the anti-HER2 A10 VHH were analyzed by FACS (filter band-pass: 564-606 nm) to assess their binding to HER2 negative (MCF10A) and positive (SKBR3) cells. Additional file 5: Table S1. Kinetic constants of fusion constructs formed by the VHH A10 and different tags for their common antigen HER2.
