# Supplementary material for: Bacterial cytoplasm as an effective cell compartment for producing functional VHH-based affinity reagents and Camelidae IgG-like recombinant antibodies
Source: Microb Cell Fact. 2014 Sep 16;13:140. doi: 10.1186/s12934-014-0140-1 (PMC4172947; doi:10.1186/s12934-014-0140-1)
Supplement: Additional file 1: Figure S1. — Application-friendly vectors for single-domain antibody expression. The vector set provides the possibility to fuse any antibody originated from a pHEN2 library to a double tag (6 x His plus specific tag) suitable for ad hoc applications. All the vectors have a conserved NcoI-NotI cloning site. Some tags are compatible for expression in both periplasm and cytoplasm whereas no conclusive data are available for other tags for which either cytoplasmic or periplasmic expression have been confirmed so far. SNAP (human O6-alkylguanine-DNA alkyltransferase); CLIP (modifid human O6-alkylguanine-DNA alkyltransferase); HALO (modified haloalkane dehalogenase); GFP (green fluorescent protein); SORT (amino acid sequence LPTEG for sortase-mediated covalent binding); BS (biotinylation sequence); cys (free cysteine for maleimide reactions); C-tag (amino acid sequence EPEA); Fc (Fc-domains, in our case rabbit and human subtype IgG2); toxin (any secreted toxin); AP (alkaline phosphatase); POX (peroxidase). [file 12934_2014_140_MOESM1_ESM.pptx]

## Slide 1
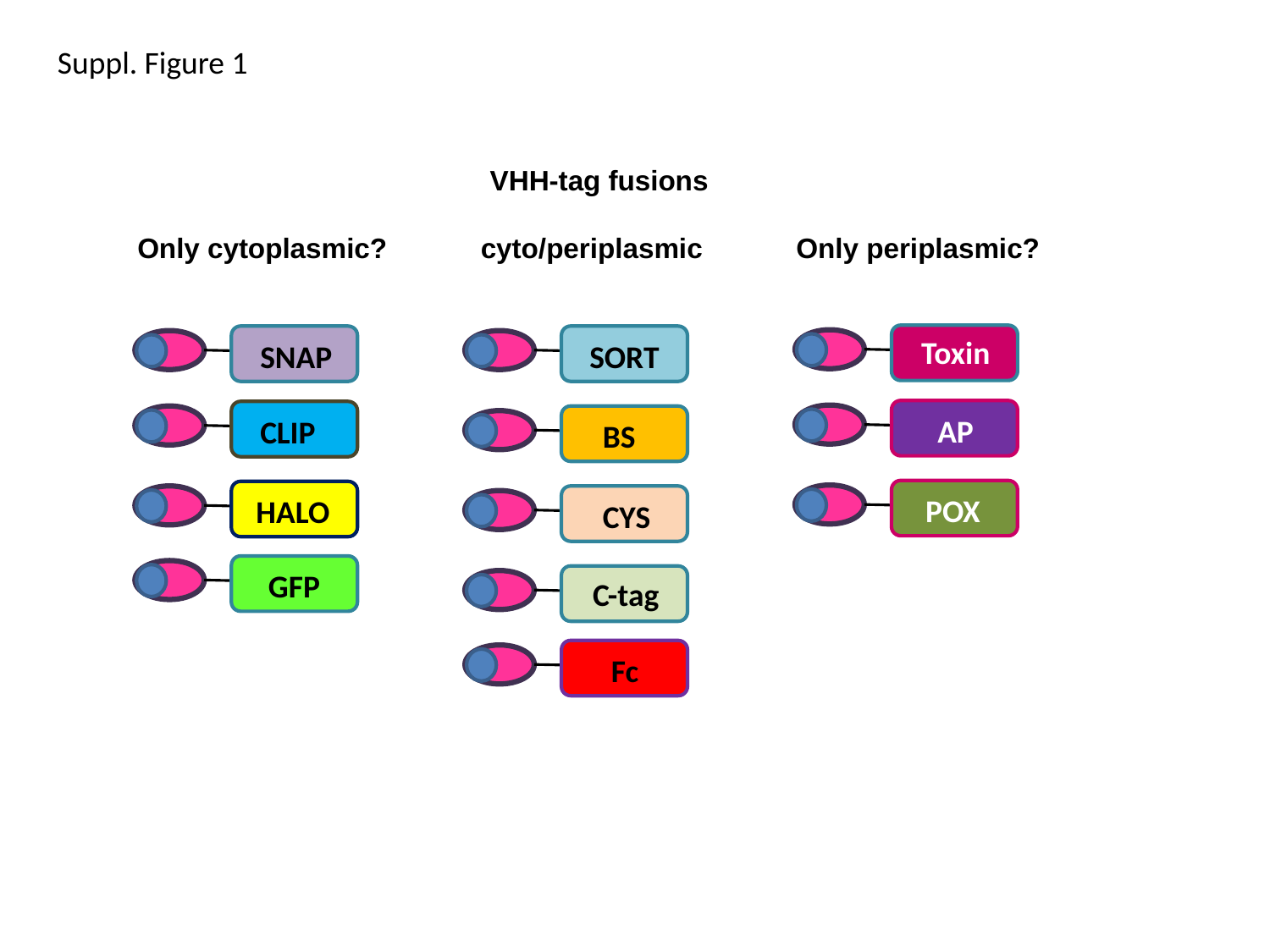

Suppl. Figure 1
 VHH-tag fusions
Only cytoplasmic? cyto/periplasmic Only periplasmic?
Toxin
SORT
SNAP
AP
CLIP
BS
POX
HALO
CYS
GFP
C-tag
Fc
