# Supplementary material for: Bacterial cytoplasm as an effective cell compartment for producing functional VHH-based affinity reagents and Camelidae IgG-like recombinant antibodies
Source: Microb Cell Fact. 2014 Sep 16;13:140. doi: 10.1186/s12934-014-0140-1 (PMC4172947; doi:10.1186/s12934-014-0140-1)
Supplement: Additional file 2: Figure S2. — Production flow-chart for the different recombinant antibody constructs. According to the vector used for cloning, the plasmids conceived for producing tagged antibodies are transformed in E. coli strains with suitable features and the corresponding antibodies will accumulate either in the bacterial cytoplasm or periplasm. Lack of transformation can be the consequence of poor cell competence or misuse of the selection antibiotics (ABs). Colonies of transformed cells are used for both pre-culture inoculation and the preparation of glycerol stocks. Cytoplasmic expression is performed in bacteria co-expressing sulfhydryl oxidase and DsbC isomerase under the control of arabinose and the production of these folding-support enzymes is triggered before the IPTG-dependent expression of the target antibody. Periplasmic expression is performed in shorter time to prevent excessive product leakage into the culture medium. The addition of glucose is intended to suppress basal expression and high IPTG concentrations should enable rapid saturation of the expression machinery. [file 12934_2014_140_MOESM2_ESM.pptx]

## Slide 1
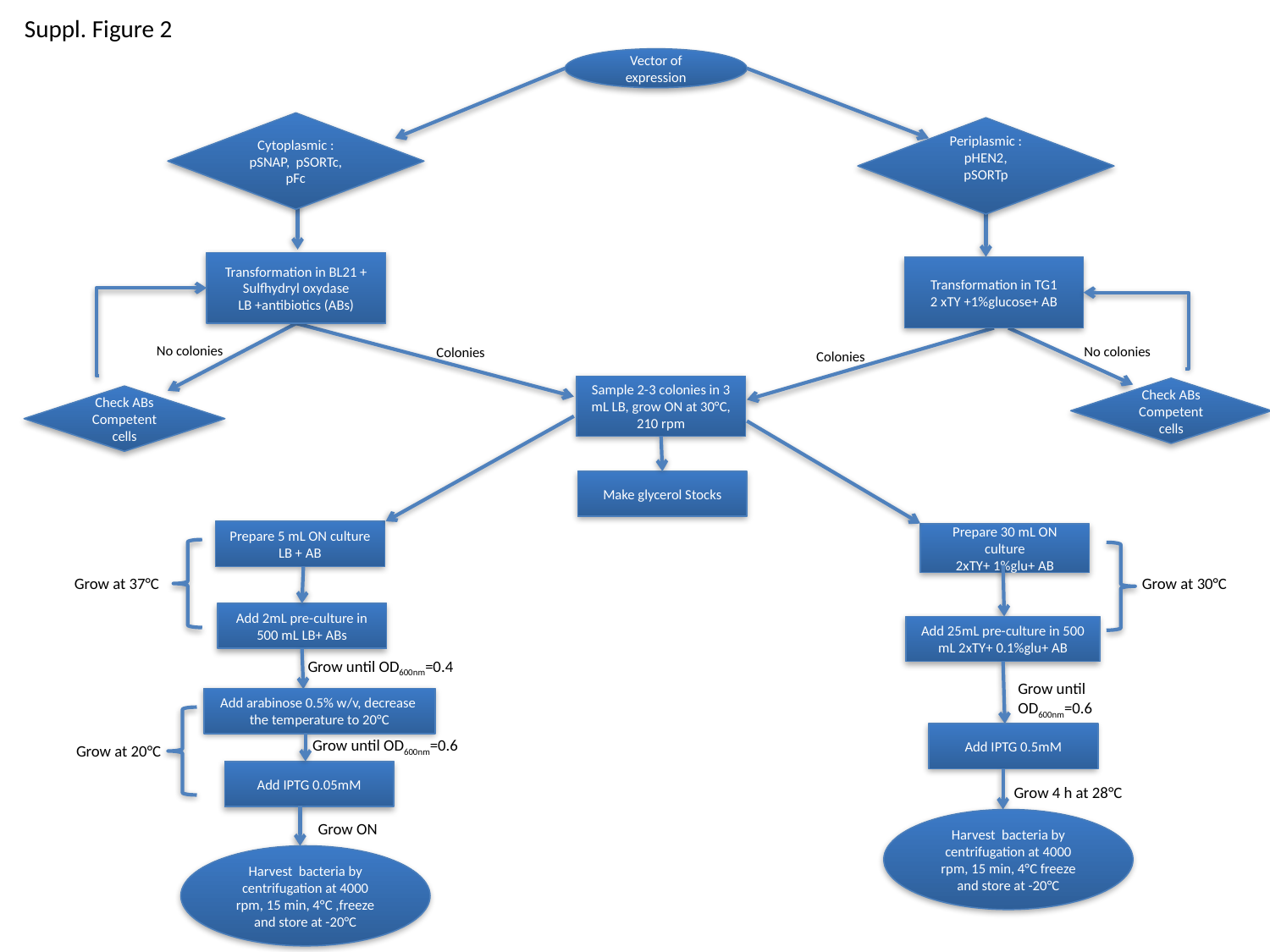

Suppl. Figure 2
Vector of expression
Cytoplasmic : pSNAP, pSORTc, pFc
Periplasmic : pHEN2,
pSORTp
Transformation in BL21 + Sulfhydryl oxydase
LB +antibiotics (ABs)
Transformation in TG1
2 xTY +1%glucose+ AB
No colonies
No colonies
Colonies
Colonies
Sample 2-3 colonies in 3 mL LB, grow ON at 30°C, 210 rpm
Check ABs
Competent cells
Check ABs
Competent cells
Make glycerol Stocks
Prepare 5 mL ON culture
LB + AB
Prepare 30 mL ON culture
2xTY+ 1%glu+ AB
Grow at 30°C
Grow at 37°C
Add 2mL pre-culture in 500 mL LB+ ABs
Add 25mL pre-culture in 500 mL 2xTY+ 0.1%glu+ AB
Grow until OD600nm=0.4
Grow until OD600nm=0.6
Add arabinose 0.5% w/v, decrease the temperature to 20°C
Add IPTG 0.5mM
Grow until OD600nm=0.6
Grow at 20°C
Add IPTG 0.05mM
Grow 4 h at 28°C
Harvest bacteria by centrifugation at 4000 rpm, 15 min, 4°C freeze and store at -20°C
Grow ON
Harvest bacteria by centrifugation at 4000 rpm, 15 min, 4°C ,freeze and store at -20°C
