# Supplementary material for: Bacterial cytoplasm as an effective cell compartment for producing functional VHH-based affinity reagents and Camelidae IgG-like recombinant antibodies
Source: Microb Cell Fact. 2014 Sep 16;13:140. doi: 10.1186/s12934-014-0140-1 (PMC4172947; doi:10.1186/s12934-014-0140-1)
Supplement: Additional file 3: Figure S3. — Quality control of the SNAP- and Fc-tagged anti-HER2 VHHs produced in the cytoplasm. SNAP-tagged antibodies were first affinity-purified by IMAC and their polydispersity was successively evaluated by analytical Size Extrusion Chromatography (SEC). The black bar corresponds to the exclusion volume. The proteins recovered in the fractions were finally separated by SDS-PAGE and visualized using colloidal blue. The A10 construct has been reported as an example.a) Fc-tagged antibodies were first expressed and purified in small-scale to assess the protein accumulation in the lysates (L) and bound to the beads after washing (B). Numbering indicates either contemporary expression induction of Fc-VHH and sulfhydryl oxidase (1) or to anticipated sulfhydryl oxidase expression (2). For large-scale production, the constructs were affinity-purified using Protein A and their polydispersity was successively evaluated by analytical SEC. The black bar corresponds to the exclusion volume. The protein was visualized after SDS-PAGE separation. The A10 and C8 constructs have been reported as examples. [file 12934_2014_140_MOESM3_ESM.pptx]

## Slide 1
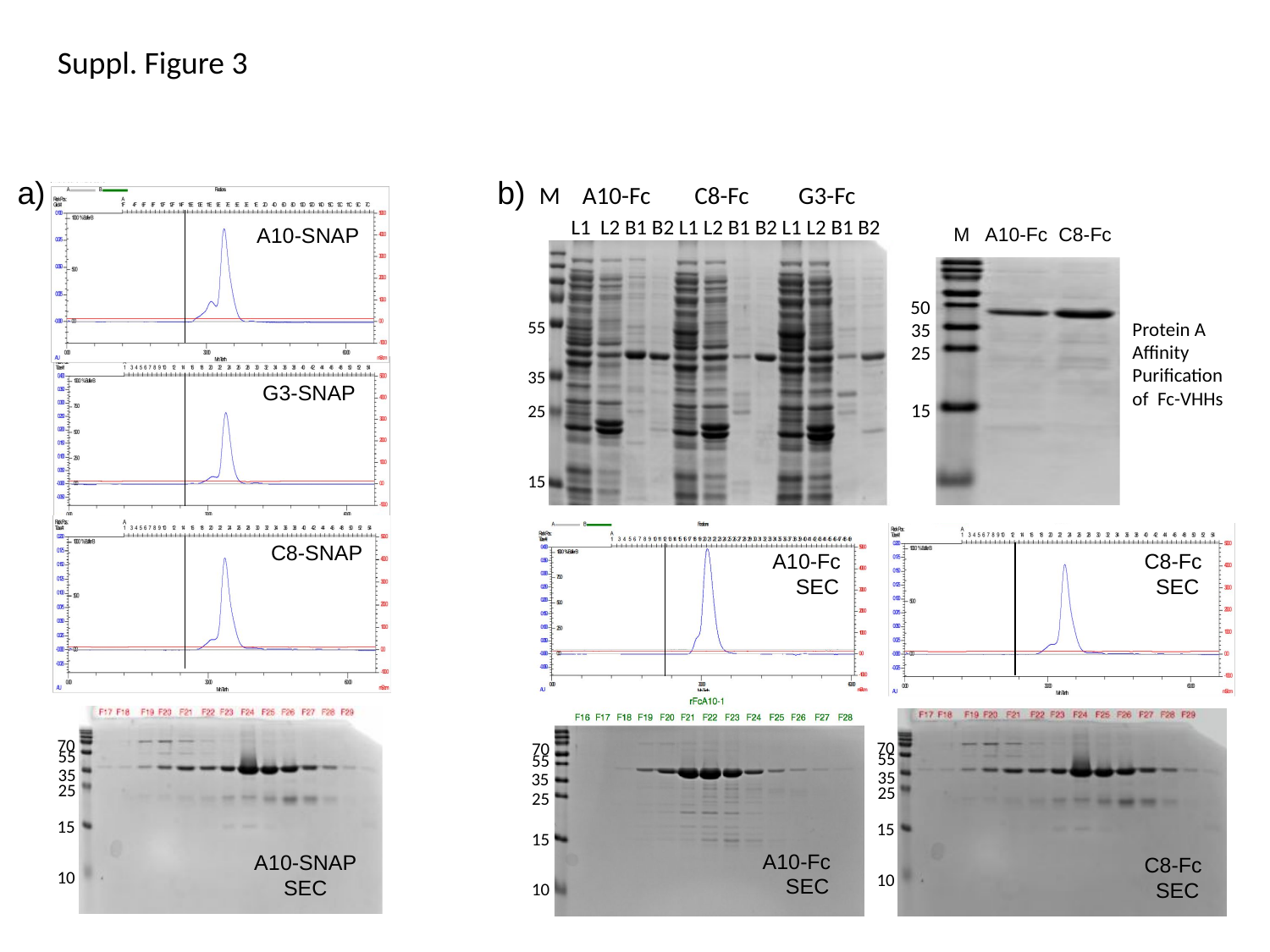

Suppl. Figure 3
a)
b)
M A10-Fc C8-Fc G3-Fc
L1 L2 B1 B2 L1 L2 B1 B2 L1 L2 B1 B2
55
35
25
15
A10-SNAP
 G3-SNAP
C8-SNAP
 M A10-Fc C8-Fc
50
35
25
15
Protein A
Affinity
Purification of Fc-VHHs
A10-Fc
SEC
 C8-Fc
SEC
A10-Fc
SEC
70
55
35
25
15
10
70
55
35
25
15
10
70
55
35
25
15
10
A10-SNAP SEC
 C8-Fc
SEC
