# Supplementary figures and images for: Bacterial cytoplasm as an effective cell compartment for producing functional VHH-based affinity reagents and Camelidae IgG-like recombinant antibodies
Source: Microb Cell Fact. 2014 Sep 16;13:140. doi: 10.1186/s12934-014-0140-1 (PMC4172947; doi:10.1186/s12934-014-0140-1)

## Slide 1
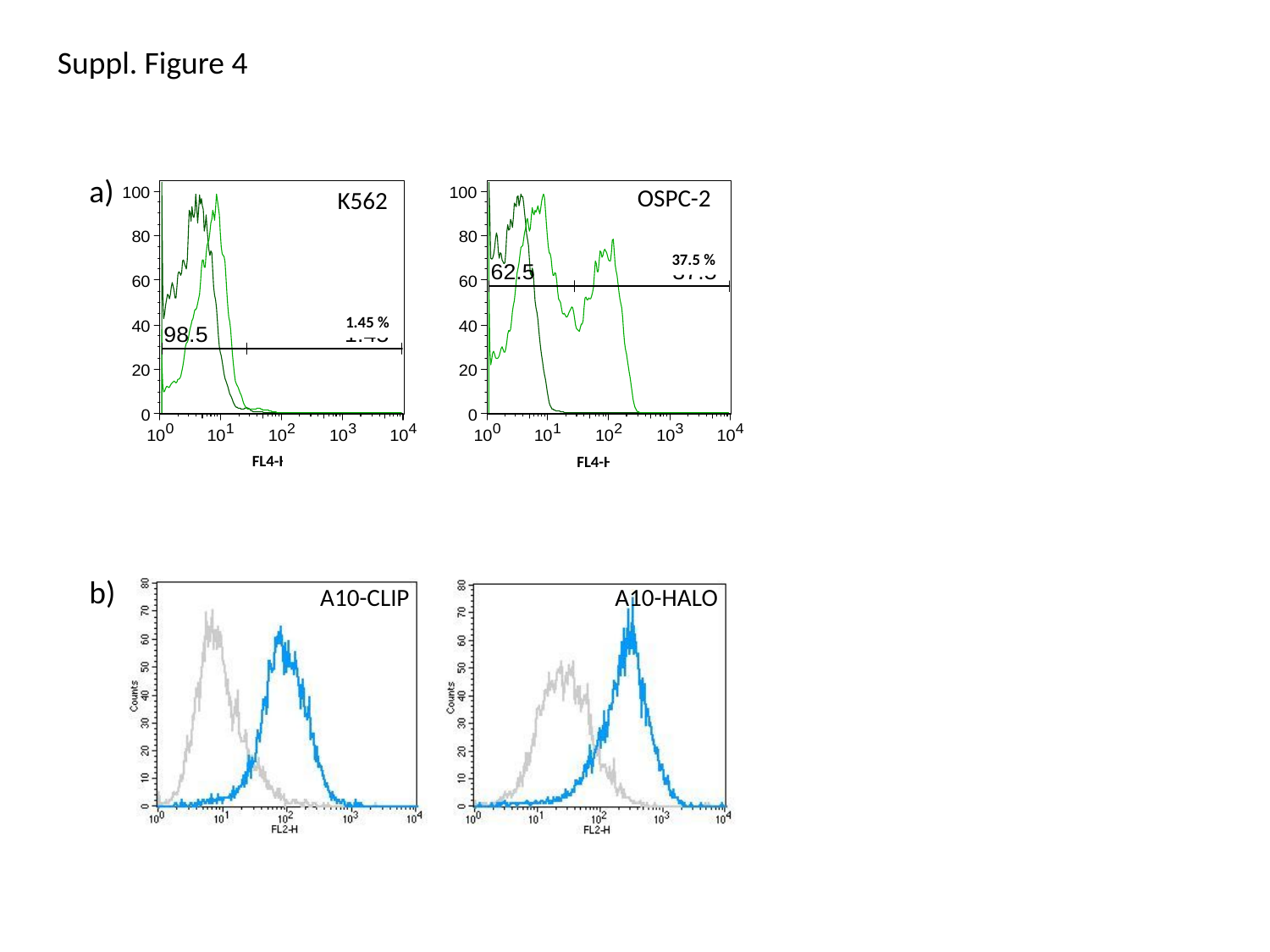

Suppl. Figure 4
K562
OSPC-2
37.5 %
FL4-H: scFvH6-Alexa647
1.45 %
FL4-H: scFvH6-Alexa647
a)
OSPC-2
K562
b)
A10-CLIP
A10-HALO

Supplement: Additional file 4: Figure S4. — Expression of fusion immunoreagents composed of antibody fragments and SNAP, CLIP, and HALO tags. a) The H6 scFv antibody specific for claudin-3 was fused to the SNAP-tag and the resulting construct labeled with Alexa 647 before FACS analysis (filter band-pass: 653-669 nm) using unfixed human ovary cancer line OSPC-2 that expresses claudin-3 and K562 cells as a negative control. Green lines indicate the level of reactivity of the scFvH6-SNAP, gray lines represent cells in the absence of the antibody. Percentage of claudin-3 postive cells is reported in each plot. b) Fusions of CLIP and HALO tags with the anti-HER2 A10 VHH were analyzed by FACS (filter band-pass: 564-606 nm) to assess their binding to HER2 negative (MCF10A) and positive (SKBR3) cells. [file 12934_2014_140_MOESM4_ESM.pptx]
