# Supplementary material for: Bacterial cytoplasm as an effective cell compartment for producing functional VHH-based affinity reagents and Camelidae IgG-like recombinant antibodies
Source: Microb Cell Fact. 2014 Sep 16;13:140. doi: 10.1186/s12934-014-0140-1 (PMC4172947; doi:10.1186/s12934-014-0140-1)
Supplement: Additional file 5: Table S1. — Kinetic constants of fusion constructs formed by the VHH A10 and different tags for their common antigen HER2. [file 12934_2014_140_MOESM5_ESM.docx]

|  | | **Kinetic constants** | | |
| --- | --- | --- | --- | --- |
| **Constructs** | **Mass (kDa)** | ***K*_D_** M | ***k*_ass_** 1/Ms | ***k*_diss_** 1/s |
| A10-myc | 15 | 1.22 x 10^-8^ | 6.88 x 10^5^ | 8.41 x 10^-3^ |
| A10-SNAP | 35 | 1.65 x 10^-8^ | 4.01 x 10^4^ | 6.60 x 10^-4^ |
| A10-SORT | 14 | 1.68 x 10^-9^ | 4.95 x 10^6^ | 8.29 x 10^-3^ |
| A10-Fc | 82 | 1.30 x 10^-10^ | 6.30 x 10^6^ | 8.21 x 10^-4^ |
| A10-AP | 130 | 1.08 x 10^-9^ | 5.88 x 10^5^ | 6.34 x 10^-4^ |
| trastuzumab | 150 | 3.03 x 10^-11^ | 1.09 x 10^6^ | 3.30 x 10^-5^ |

**Additional file 5: Table S1**. Kinetic constants of fusion constructs formed by the VHH A10 and different tags for their common antigen HER2
